# INTERNALIZED AGEISM AS A RISK FACTOR FOR SUICIDAL IDEATION IN LATER LIFE

**DOI:** 10.1093/geroni/igae098.0015

**Published:** 2024-12-31

**Authors:** Tracey Gendron, Alyssa Camp, Amateau Gigi

**Affiliations:** Virginia Commonwealth University, Richmond, Virginia, United States; Virginia Commonwealth University, Richmond, Virginia, United States; Virginia Commonwealth University, Richmond, Virginia, United States

## Abstract

The Interpersonal Theory of Suicide proposes that suicidal ideation is based on the immutability of two constructs – perceived burdensomeness and thwarted belongingness. This study examined the influence of ageism on suicidal ideation using the framework of the Interpersonal Theory of Suicide, investigating whether internalized and relational ageism contribute to suicidal ideation beyond the variance already explained by demographic factors, thwarted belonging, and perceived burdensomeness. Using Research Match volunteers 65 and older in the United States were recruited to participate in an online survey. A total of 454 individuals from over 30 states participated in the study. Twelve percent of respondents reported that they had experienced suicidal ideation in the past month. Hierarchical Logistic Regression analysis demonstrated that age (OR = 1.07, 95% CI [1.00, 1.15]), and internalized ageism (OR = 1.19, 95% CI [1.07, 1.32]) had significant associations with suicidal ideation. Among all independent variables, internalized ageism had the strongest association with suicidal ideation. Ageism can result in people feeling their needs being ignored or dismissed due to their age or feeling isolated from a world that does not recognize or value their contributions. Findings from this study further amplify the critical importance of understanding the real-world impact of ageism.

